# Deduction of Novel Genes Potentially Involved in Keratinocytes of Type 2 Diabetes Using Next-Generation Sequencing and Bioinformatics Approaches

**DOI:** 10.3390/jcm8010073

**Published:** 2019-01-10

**Authors:** En-Shyh Lin, Wei-An Chang, Yang-Yi Chen, Ling-Yu Wu, Yi-Jen Chen, Po-Lin Kuo

**Affiliations:** 1 Department of Beauty Science, National Taichung University of Science and Technology, Taichung 403, Taiwan; eslin7620@gmail.com; 2 Graduate Institute of Clinical Medicine, College of Medicine, Kaohsiung Medical University, Kaohsiung 807, Taiwan; 960215kmuh@gmail.com (W.-A.C.); 1050556@kmuh.org.tw (Y.-Y.C.); esther906@gmail.com (L.-Y.W.); 3 Division of Pulmonary and Critical Care Medicine, Kaohsiung Medical University Hospital, Kaohsiung 807, Taiwan; 4 Department of Dermatology, Kaohsiung Medical University Hospital, Kaohsiung 807, Taiwan; 5 Department of Physical Medicine and Rehabilitation, Kaohsiung Medical University Hospital, Kaohsiung 807, Taiwan; 6 Institute of Medical Science and Technology, National Sun Yat-Sen University, Kaohsiung 804, Taiwan

**Keywords:** type 2 diabetes, keratinocytes, immune response, next-generation sequencing, bioinformatics, microRNA, messenger RNA

## Abstract

Keratinocytes constitute the major cell type of epidermis, which participates in re-epithelialization during wound repair and the immune defense response to pathogens. The aim of the current study was to explore the differentially expressed genes and novel microRNA (miRNA) regulations that are potentially involved in diabetic keratinocytes through next-generation sequencing (NGS) and bioinformatics approaches. A total of 420 differentially expressed genes between normal and diabetic keratinocytes were identified, and systematic bioinformatics analyses indicated that these differentially expressed genes were functionally enriched in interferon-alpha signaling, viral defense response, and immune response. Additionally, the potential miR-340-3p-*DTX3L* interaction that has been systematically validated in miRNA prediction databases was proposed to participate in the disrupted skin homeostasis, altering the defense and immune response of diabetic skin. The findings may provide new insights into understanding the pathogenesis of epidermal pathologies in diabetic patients and targeting novel molecules to advance diabetic skin care in clinical practice.

## 1. Introduction

The global prevalence of diabetes mellitus is approximately 9%, and type 2 diabetes (T2D) accounts for 90% of all diabetes. The rapidly increasing prevalence has made diabetes a major public health concern [1,2]. Patients with T2D have increased risks of both macrovascular and microvascular complications, which are the primary causes of morbidity and mortality in T2D [2]. The high prevalence of skin disorders related to impaired glycemic control and vascular complications has been reported, while early-stage skin disorders are often overlooked, and may result in ulcers and skin and soft tissue infections in patients with T2D [3]. The T2D population is also susceptible to poor wound healing, which has been proposed to be associated with poor glycemic control and the accumulation of advanced glycation end products (AGEs) in the skin [3,4,5].

Human skin consists of epidermal and dermal layers. Unlike the dermis layer having greater cell diversity, epidermis consists majorly of keratinocytes, which are responsible for re-epithelialization by interacting with fibroblasts in a paracrine manner during the wound-healing process [6,7]. A high-glucose environment and increased AGEs have been suggested to impair keratinocyte proliferation and differentiation, and inhibit the migratory ability of keratinocytes, suggesting the essential role of keratinocytes in diabetic wound healing [8,9,10]. Recent evidence has also suggested the key role of genetic and epigenetic regulation in diabetic foot ulcer and the wound-healing process [11,12]. MicroRNAs (miRNAs) negatively regulate the expression of many human genes through 3’-untranslated region (UTR) binding at the post-transcriptional level, and provide the fine-tuning of gene regulations [13]. Recent studies have suggested the essential role of miRNA regulation in the biological processes of various skin cell types [12,14], including immune cells, fibroblasts, and keratinocytes, and dysregulated miRNAs in diabetic skins participate in the impaired wound healing [15,16,17,18,19]. The importance of miRNA regulation in diabetic wound healing has been emphasized, thus prompting the development of topical miRNA-directed therapy for diabetic skin care [20,21].

Recent advances in the deep sequencing of the whole genome has provided high-throughput genomic profiling and facilitated the development of individualized precision medicine [22,23], and abundant software tools for an integrative analysis of the big data are available, which can enable more efficiently gaining a comprehensive understanding of the biological functions of a list of genes [24,25,26]. In the current study, we aimed to investigate the differentially expressed genes and novel miRNA regulations that are potentially involved in diabetic keratinocytes, using next-generation sequencing (NGS) and bioinformatics approaches. We hope that the findings will provide novel insights into understanding the underlying pathogenesis of epidermal pathologies in patients with T2D.

## 2. Materials and Methods

### 2.1. Experimental Design

**Figure 1:**
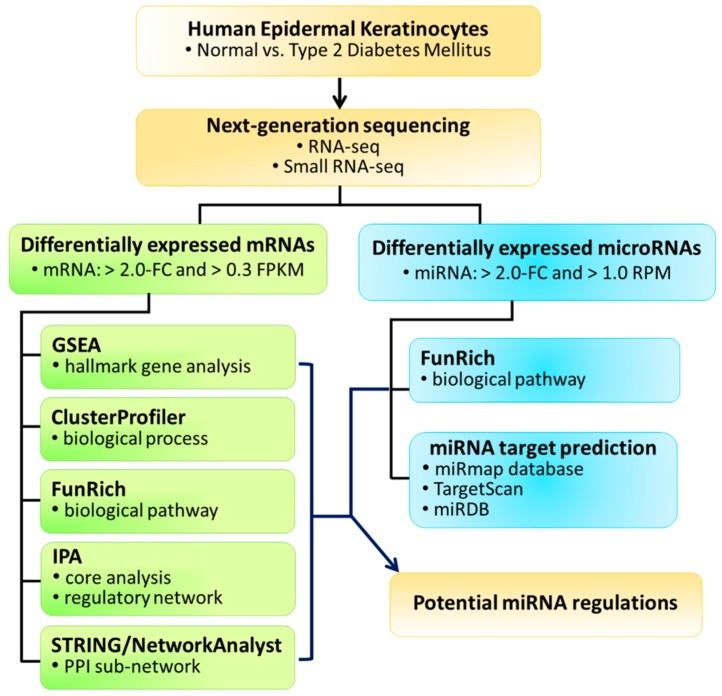
Study flowchart. Human epidermal keratinocytes of normal and type 2 diabetic skin were deep sequenced for RNA and small RNA expression profiles, and systematically analyzed using bioinformatics databases for deduction of novel molecular signatures in diabetic keratinocytes. FC, fold-change; FPKM, fragments per kilobase of transcript per million mapped reads; RPM, reads per million; PPI, protein-protein interaction; GSEA, gene set enrichment analysis; IPA, Ingenuity Pathway Analysis; STRING, Search Tool for the Retrieval of Interacting Genes/Proteins.

To explore the differential expression profiles between normal and T2D epidermal keratinocytes, RNAs were extracted from human epidermal keratinocytes of normal (N-HEK) and type 2 diabetes mellitus (DM-HEK) origins for next-generation sequencing (NGS) and further systematic bioinformatics analysis. The study flowchart is provided in Figure 1.

### 2.2. Cell Culture of Primary Human Epidermal Keratinocytes

Human adult N-HEK and DM-HEK cells were purchased from Lonza Walkersville Inc. (Walkersville, MD, USA) and cultured in keratinocyte serum-free growth medium (Cell Applications, Inc. San Diego, CA, USA) according to recommended culturing protocol in 5% CO_2_ humidified incubator at 37 °C. After growth to confluence, adult N-HEK and DM-HEK cells were harvested for further RNA extraction using Trizol Reagent (Invitrogen, Carlsbad, CA, USA), following the manufacturer’s instructions, and tested for the quality of extracted RNAs. The OD_260_/OD_280_ absorbance ratio detected by ND-1000 spectrophotometer (Nanodrop Technology, Wilmington, DE, USA) was 2.03 for N-HEK and 2.04 for DM-HEK, and the RNA integrity number (RIN) determined by Bioanalyzer 2100 (Agilent Technology, Santa Clara, CA, USA) was 9.9 for both N-HEK and DM-HEK, indicating the good quality of the extracted RNAs.

### 2.3. Next-Generation Sequencing

The extracted total RNAs of adult N-HEK and DM-HEK cells were sequenced for RNA and small RNA expression profiles by Welgene Biotechnology Company (Welgene, Taipei, Taiwan). Briefly, all of the RNA samples were prepared following the Illumina protocol, and Agilent’s SureSelect Strand-Specific RNA Library Preparation kit was used for RNA library construction, followed by AMPure XP Beads size selection. The RNA sequencing was determined by Illumina’s sequencing-by-synthesis technology, with a read length of 150 nucleotides per pair-end, and the sequencing data was generated by Welgene’s pipeline based on Illumina’s base-calling program bcl2fastq v2.2.0. The raw reads were trimmed to remove lower quality bases using Trimmomatic (version 0.36). The passed reads were then aligned to a reference human genome sequence obtained from the Ensembl database (GRCh38.p12) using the HISAT2 alignment tool. The expression value of each gene was normalized by calculating the fragments per kilobase of transcript per million mapped reads (FPKM). The differential expression analysis between N-HEK and DM-HEK cells was carried out using Cuffdiff (Cufflinks version 2.2.1). For small RNA sequencing, the library construction was prepared using an Illumina sample preparation kit, which contained 3′ and 5′ adaptors that were ligated to total RNA, reverse transcribed to cDNA, fractionated, and purified for bands with 18–40 nucleotide fragments. The sequencing for a single-end read length of 75 nucleotides was carried out using the Illumina instrument and software, in which raw sequences were trimmed for qualified reads, analyzed in miRDeep2 to clip 3’ adaptor sequence, and aligned to a reference human genome from the University of California, Santa Cruz (UCSC). The miRDeep2 was used for the detection of known miRNAs and their estimated expression values were normalized in reads per million (RPM).

### 2.4. clusterProfiler

The clusterProfiler is an ontology-based tool implemented in R, which is an open-source programming environment, to analyze functional profiles in gene ontology (GO) terms and the Kyoto Encyclopedia of Genes and Genomes (KEGG) pathways of genes and/or gene clusters for a functional enrichment test based on hypergeometric distribution. The adjusted *p*-values were estimated to prevent a high false discovery rate (FDR) in multiple testing [27]. The clusterProfiler version 3.6 was used for the analysis, and the differentially expressed genes in DM-HEK cells were analyzed for functional profiles.

### 2.5. Gene Set Enrichment Analysis (GSEA)

The Gene Set Enrichment Analysis (GSEA) software provides a powerful analytical method to interpret large gene expression data. The analysis is based on biologically defined gene sets consisted of groups of genes with common biological function, regulation, or chromosomal location. GSEA considers all of the expressed genes in an experiment, instead of selected genes with significant differential expression, and then assesses the significance by permuting phenotypes, which can preserve gene–gene correlations. Moreover, a leading-edge analysis is provided to help define core genes that are representative of biological importance within the gene set [28]. The GSEA desktop version 3.0 was used for the analysis.

### 2.6. Functional Enrichment Analysis Tool (FunRich)

FunRich is an open access, Windows-based analytic tool for both the functional enrichment and interaction network analysis of genes and proteins. The FunRich analysis is based on a backend database with the human-specific collated genomic and proteomic datasets of more than 1.5 million annotations, which were regularly updated. The statistical significance of enriched terms is performed by a hypergeometric distribution test, and the FDR method is also implemented to correct for multiple testing. The results can be graphically presented in customized forms [29,30]. The FunRich version 3.1.3 was used for the analysis.

### 2.7. STRING and NetworkAnalyst Databases for Protein–Protein Interaction Network Analysis Database

The STRING database collects and integrates all of the functional interactions between expressed proteins from known and predicted protein–protein association data. The latest version of STRING covers 2031 organisms: 9.6 million proteins and 1380 million interactions. The differentially expressed genes were uploaded for the protein–protein interaction (PPI) network, and interactions with at least medium confidence (interaction score > 0.4) were set by default. The STRING database also provides enrichment analysis for various classification systems, including GO and KEGG, and implements Fisher’s exact test followed by correction for multiple testing to determine statistical significance [31]. The retrieved large PPI network was uploaded into Cytoscape software package with Molecular Complex Detection (MCODE) plugin tool to analyze the clusters of PPI sub-networks [32]. The STRING version 10.5 and Cytoscape version 3.6.1 were used for the analysis.

NetworkAnalyst is a web-based tool supporting the network-based meta-analysis of gene expression data in an integrative approach. A gene list of interest was uploaded and mapped to the manually curated International Molecular Exchange (IMEx) PPI database, and the minimum interaction network type selected to construct the relevant networks [33,34].

### 2.8. Ingenuity Pathway Analysis (IPA)

IPA is a software package (Ingenuity systems, Redwood City, CA, USA) that is available for the bioinformatics analysis of genomic, proteomic, and experimental studies based on curated literature searches reviewed and updated by experts. The gene list with expression changes is uploaded to IPA software for core analysis to obtain predicted canonical pathways, associated diseases and functions, and interaction networks [35]. In addition, IPA also provides causal analytics tools for the construction of causal networks to generate mechanistic hypotheses based on the directional changes of expression in the uploaded dataset. The statistical significance of the enriched functions or constructed networks was determined by two scores: the enrichment score (Fisher’s exact test *p*-value), which assesses the overlap of observed and predicted gene sets, and the *z*-score, which assesses the match of observed and predicted regulatory patterns with a prediction for the activation state [36]. In the current study, differentially expressed genes in DM-HEK cells with fold-changes in expression were uploaded for the core analysis and construction of the causal network.

### 2.9. miRmap Target Prediction Database

The miRmap is an open-source software library for the target prediction of a specific miRNA. It is the first miRNA target prediction tool that comprehensively covers different approaches for the prediction of repression strength, including probabilistic, evolutionary, thermodynamic, and sequence-based approaches. The repression strength is indicated as a miRmap score, with a higher score representing a higher repression strength [37]. The miRmap version 1.0 was used in the current study to predict the putative targets of differentially expressed miRNAs in DM-HEK cells.

## 3. Results

### 3.1. Identification of Differentially Expressed Genes in Human Type 2 Diabetic Epidermal Keratinocytes (DM-HEK)

**Figure 2:**
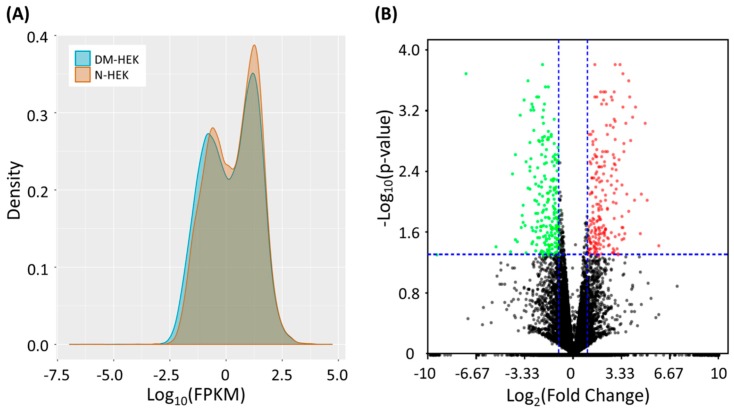
Differential expression patterns of normal (N-HEK) and diabetic (DM-HEK) human epidermal keratinocytes from next-generation sequencing (NGS) displayed in (**A**) a density plot and (**B**) a volcano plot. The dots in red represent significantly up-regulated genes, and the dots in green represent significantly down-regulated genes in DM-HEK cells. DM-HEK, diabetic human epidermal keratinocytes; N-HEK, normal human epidermal keratinocytes.

The expression profiling of adult N-HEK and DM-HEK cells were retrieved from NGS results, and the expression values were normalized in FPKM. The distribution of the FPKM values of the two samples were displayed in a density plot (Figure 2A). The differentially expressed genes in DM-HEK cells were screened by the following selection criteria: expression values of higher than 0.3 FPKM in either sample, at least a 2.0-fold-change between N-HEK and DM-HEK cells, and a significant between-sample differential expression with a *p*-value < 0.05. The distribution of differentially expressed genes between N-HEK and DM-HEK cells were plotted in a volcano plot, as shown in Figure 2B. The selection criteria yielded a total of 420 differentially expressed genes, with 209 up-regulated genes and 211 down-regulated genes in DM-HEK cells.

### 3.2. The Differentially Expressed Genes in DM-HEK Were Enriched in Interferon (IFN) Signaling and Viral Defense Response

**Figure 3:**
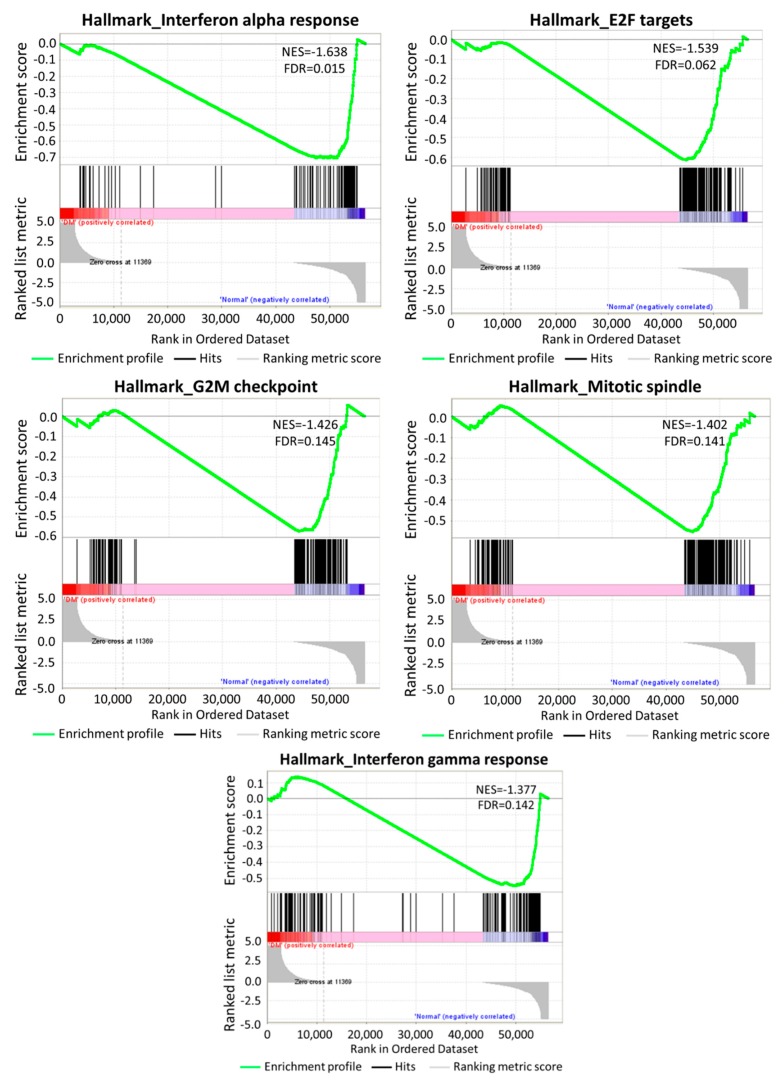
Gene Set Enrichment Analysis (GSEA) of gene expressions in keratinocytes. All of the expressed genes in N-HEK and DM-HEK cells were uploaded into GSEA for enrichment analysis. The h.all.v6.2.symbols.gmt [Hallmarks] gene set database was used as the gene set collection for analysis. GSEA performed 1000 permutations. The maximum and minimum sizes for gene sets were set at 500 and 15, respectively. Cutoff for significant gene sets was false discovery rate <25%, by default. NES, normalized enrichment score; FDR, false discovery rate.

The differentially expressed genes were systematically analyzed in the following bioinformatics databases for functional enrichment analysis, including GSEA, clusterProfiler, and FunRich. Firstly, the expression values of all of the genes in N-HEK and DM-HEK cells were uploaded into GSEA software to analyze the enriched functions within the hallmark gene sets database. The default cutoff for the significantly enriched gene sets was set at a FDR of <25%. The results identified that the gene sets that were related to interferon alpha/gamma response and cell cycle function were significantly enriched in N-HEK cells, as shown in Figure 3.

**Figure 4:**
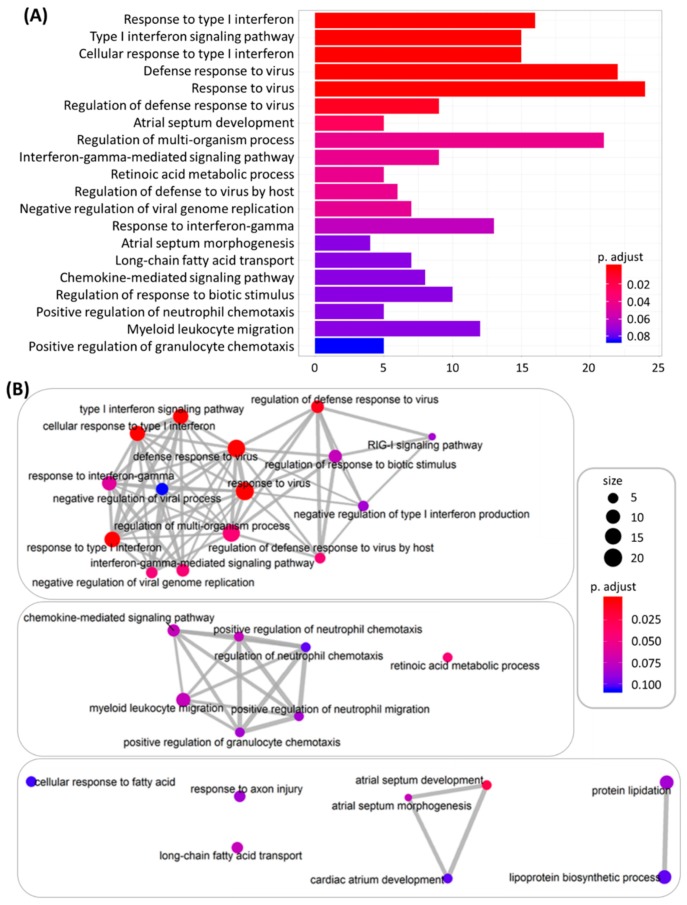
(**A**) Top 20 functionally enriched biological processes with corresponding adjusted *p*-values analyzed by clusterProfiler, which are displayed in a bar chart. The color scales indicated the different thresholds of adjusted *p*-values. (**B**) Interaction networks between enriched biological processes analyzed by enrichMap in the clusterProfiler package. The color scales indicated different thresholds of adjusted *p*-values, and the sizes of the dots represented the percentage of each term.

**Figure 5:**
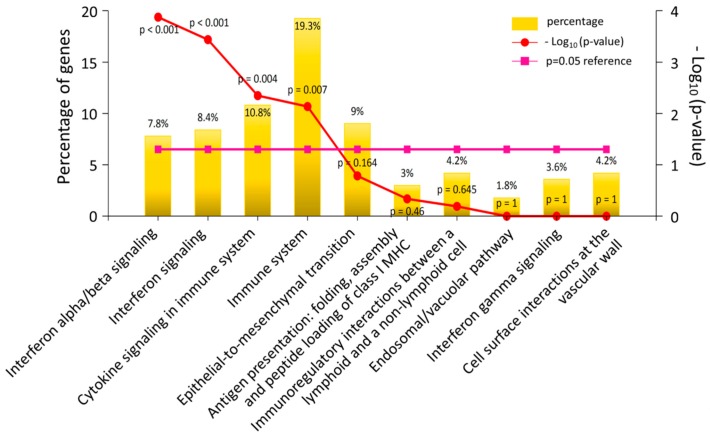
Enriched biological pathways of differentially expressed genes in diabetic keratinocytes. Top 10 biological pathways with their corresponding p-values and percentages of genes were obtained from enrichment analysis using the FunRich analytic tool, and are displayed in a bar chart. The significantly enriched biological pathways (*p* < 0.05) included interferon signaling and cytokine signaling in the immune system.

In addition, the 420 differentially expressed genes were input into the clusterProfiler and FunRich databases for functional enrichment analysis. The top 20 functionally enriched biological processes with their corresponding adjusted *p*-values obtained from clusterProfiler analysis under GO terms are indicated in a bar chart in Figure 4A, and the most significantly enriched biological processes were associated with type I interferon (IFN) signaling and defense response to virus. The interaction networks between enriched biological processes were analyzed using the enrichMap function in the clusterProfiler package, which yielded a dense interaction network among the biological processes related to IFN signaling and defense response to virus, as shown in the upper part of the network cluster in Figure 4B. The biological pathway analysis results from the FunRich database indicated that these differentially expressed genes were significantly enriched in IFN signaling and cytokine signaling in the immune system, as shown in Figure 5. Taken together, the systematic bioinformatics analysis of the differentially expressed genes between N-HEK and DM-HEK cells were functionally enriched in IFN-alpha signaling related to the immune system. Therefore, we hypothesized that normal and DM keratinocytes exert different gene expression profiles that are related to altered defense response in skin.

### 3.3. Identification of Potential Mechanistic Regulatory Network and Gene Clusters Involved in IFN Signaling and Defense Response of HEK Cells

**Figure 6:**
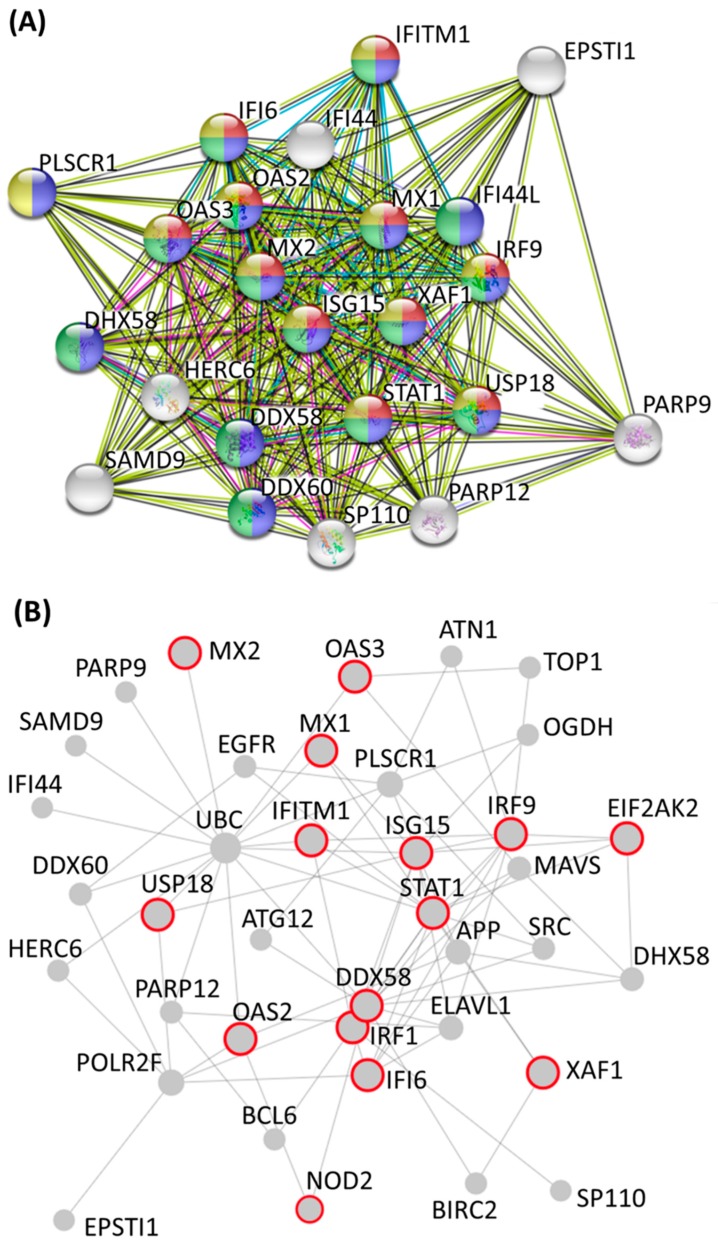
Protein–protein interaction (PPI) network in cluster 1 from the MCODE sub-network analysis. (**A**) The interaction network and functional enrichment analysis of the 23 molecules in cluster 1 were obtained from the STRING database. Molecules involved in the type I interferon signaling pathway are colored in red, the defense response-related molecules are colored in blue, the immune response-related molecules are colored in green, and the cytokine response-related molecules are colored in yellow. (**B**) The interaction network was validated using NetworkAnalyst with the International Molecular Exchange (IMEx) PPI database. The molecules that are involved in interferon signaling and cytokine signaling in the immune system are indicated in the red frame.

To further identify clusters of genes among the 420 differentially expressed genes and their associated biological functions, these genes were input into the STRING database for potential PPI networks. A large PPI network (PPI enrichment *p*-value < 1.0 *×* 10^−16^) with 414 nodes and 728 edges was retrieved, and further sub-network analysis was performed using the plug-in Molecular Complex Detection (MCODE) tool in Cytoscape. The clusters of sub-networks that were retrieved from MCODE analysis are listed in Table 1, with the 23 molecules grouped into cluster 1 showing the highest score. These 23 molecules were analyzed in the STRING database for enrichment analysis in GO terms, and the top 10 biological processes that were enriched in these genes were associated with the defense response to virus, type I IFN signaling, and related immune responses, as listed in Table 2. The PPI network among the molecules of cluster 1 was obtained from the STRING database, as presented in Figure 6A, and the molecules that were related to the biological processes of the type I IFN signaling pathway, defense response, immune response, and response to cytokine were indicated. The 23 molecules were uploaded to NetworkAnalyst for network validation using IMEx PPI database (Figure 6B).

**Figure 7:**
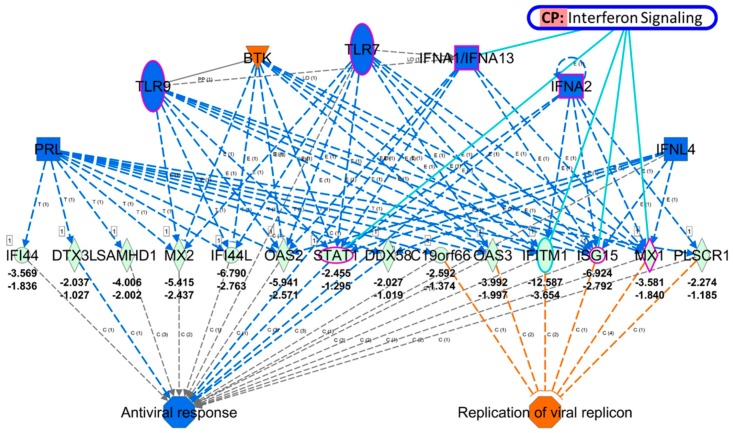
Proposed regulatory network from Ingenuity Pathway Analysis software. The proposed mechanistic network with predicted upstream regulator and associated downstream effectors and final functions were generated based on assessing the match of observed and predicted regulatory patterns. The overlay canonical pathway among the mechanistic network identified *STAT1*, *IFITM1*, *ISG15*, and *MX1* as associated with the Interferon (IFN) signaling pathway. Molecules in purple frames, including *STAT1*, *ISG15*, and *MX1* from our dataset, were associated with dermatitis and the inflammatory response. Molecules in light green indicated decreased expression, while orange indicated predicted activation, and blue indicated predicted inhibition.

To further identify the genes that are potentially involved in the defense response of HEK cells, expression values of the 420 differentially expressed genes were uploaded into IPA software for core analysis. As indicated in other bioinformatics databases, IFN signaling was one of the top canonical pathways (*p*-value = 8.79 *×* 10^−5^, *z*-score = −2.236) identified in the IPA results. Additionally, the regulator effect network retrieved from the core analysis result showed a mechanistic network (consistency score = 9.087) that was associated with functions of antiviral response (*p*-value = 9.15 *×* 10^−10^, *z*-score = −2.0) and replication of viral replicon (*p*-value = 1.2 *×* 10^−4^, *z*-score = 2.407). The proposed mechanistic network with predicted upstream regulator and associated downstream effectors and final functions is shown in Figure 7. Among the generated regulatory network, *STAT1*, *IFITM1*, *ISG15*, and *MX1* were genes associated with the IFN signaling pathway, while *STAT1* and *MX1* were associated with dermatitis, and *STAT1* and *ISG15* were associated with the inflammatory response.

### 3.4. Identification of Potential miRNA–mRNA Interactions in DM-HEK Cells

To investigate the potential miRNA regulations in DM-HEK cells, we simultaneously performed small RNA sequencing for miRNA profiling in adult N-HEK and DM-HEK cells. Differentially expressed miRNAs in DM-HEK cells were selected under the selection criteria of normalized read counts >one RPM and at least a 2.0-fold-change between N-HEK and DM-HEK cells. The selection criteria yielded 87 differentially expressed miRNAs, including 44 up-regulated and 43 down-regulated miRNAs in DM-HEK cells.

**Figure 8:**
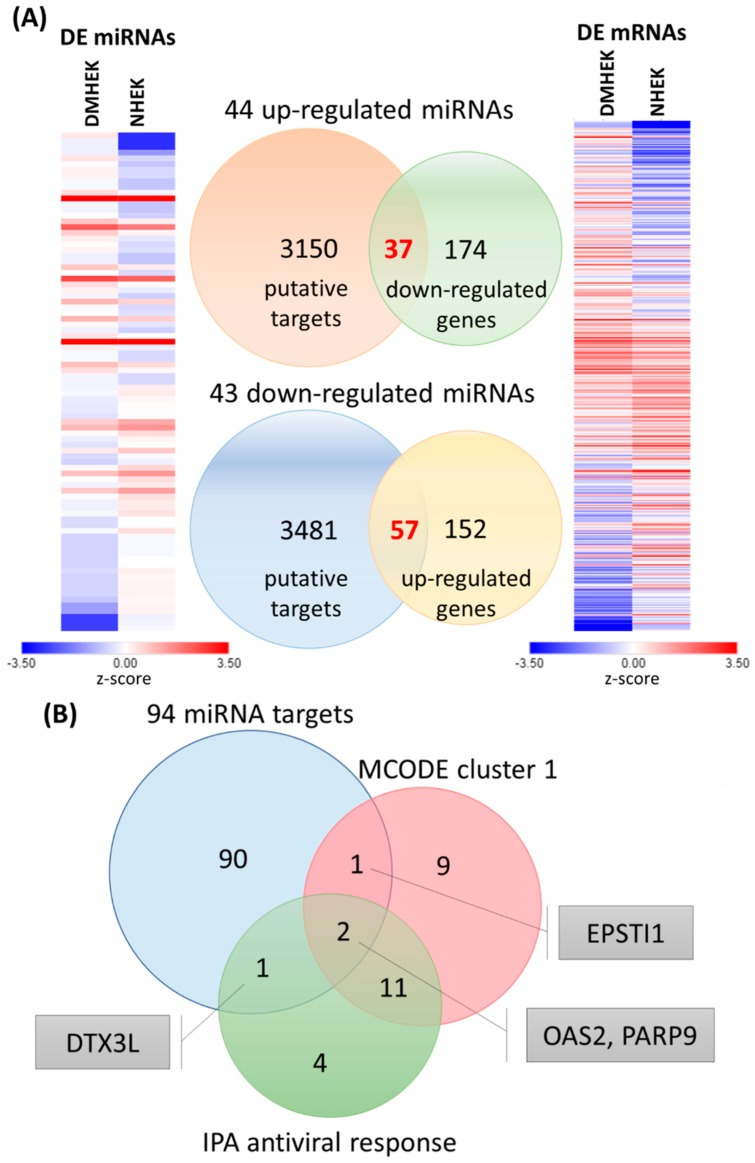
Differentially expressed miRNAs and mRNAs with potential miRNA targets and the deduction of novel genes related to defense response in diabetic keratinocytes. (**A**) The heat maps of differentially expressed miRNAs and mRNAs in N-HEK and DM-HEK cells are shown in the left and right panels, respectively. Putative miRNA targets were predicted using the miRmap database, setting the repression score at ≥97.0. The candidate genes were those overlapping with differentially expressed mRNAs in HEK cells, as shown in the Venn diagram in the middle panel. (**B**) The 94 miRNA targets were matched to 23 genes, which were identified as cluster 1 from Molecular Complex Detection (MCODE) sub-network analysis, and 18 genes related to antiviral response from the Ingenuity Pathway Analysis (IPA) result for the deduction of novel genes related to defense response in DM-HEK cells, including *DTX3L*, *EPSTI1*, *OAS2*, and *PARP9*.

Using the miRmap database, the 44 up-regulated and 43 down-regulated miRNAs in DM-HEK cells were input into the database for miRNA target prediction, and those predicted targets with miRmap scores >97.0 were selected. The selected putative targets of up-regulated (down-regulated) miRNAs were matched to our dataset of 211 down-regulated (209 up-regulated) genes in DM-HEK cells. The matched result is displayed in a Venn diagram along with heatmaps in z-score values of differentially expressed miRNAs and mRNAs in Figure 8A. A total of 94 genes with potential miRNA regulations were identified. 

To identify the overlapping genes between 94 potential miRNA targets and clusters of genes already indicated in MCODE cluster 1 and the antiviral response in the IPA result, the Venn diagram analysis was carried out, and identified four overlapping genes of interest, including *OAS2*, *PARP9*, *EPSTI1*, and *DTX3L* (Figure 8B).

**Figure 9:**
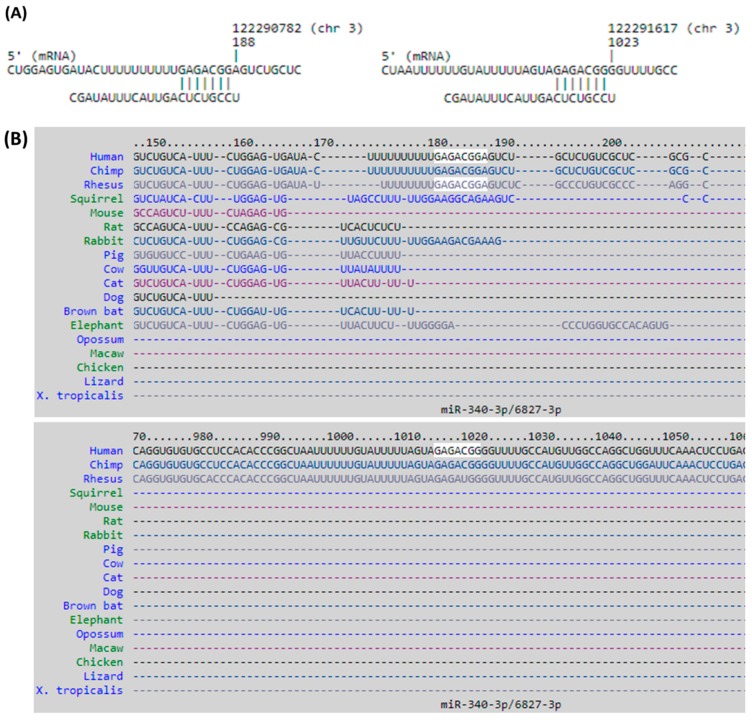

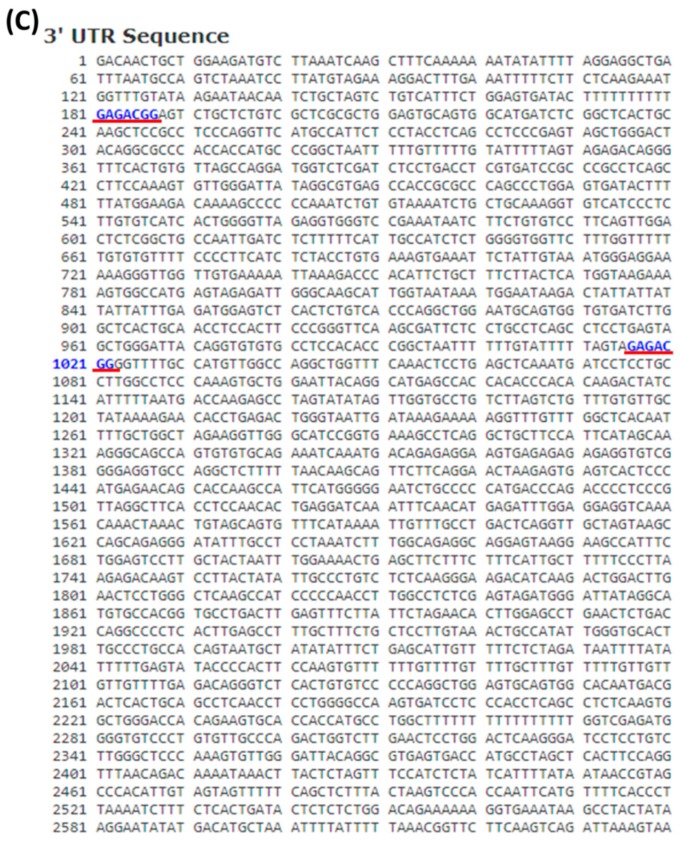
The putative binding sites of miR-340-3p on DTX3L. The sequences and putative 3′-untranslated region (UTR) binding sites of miR-340-3p on DTX3L at positions of 181–187 and 1016–1022 were validated in miRmap (**A**), TargetScan (**B**), and miRDB (**C**) databases.

The four target genes with corresponding miRNA regulations and respective miRmap scores were listed in Table 3. To validate these potential miRNA regulations systematically, the other two miRNA target prediction databases were used, including TargetScan and miRDB, and the analytic results yielded one potential miRNA regulation with consistent putative 3′-UTR binding sites at positions of 181–187 and 1016–1022 in all three miRNA prediction databases, the miR-340-3p-*DTX3L* interaction (Figure 9). 

## 4. Discussion

**Figure 10:**
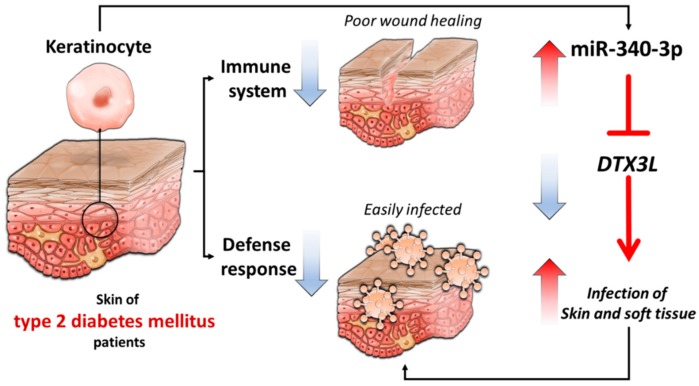
Schematic summary of proposed molecular signatures in diabetic skin pathology.

The current study identified that the differentially expressed genes in DM-HEK cells were enriched in biological functions of IFN-alpha signaling, viral defense response, and immune response, through NGS and systematic bioinformatics analysis. In addition, the potential miR-340-3p-*DTX3L* interaction validated in different miRNA prediction databases was proposed to participate in the altered defense and immune response of diabetic skin. The proposed molecular signatures in DM-HEK cells are presented in graphic summary in Figure 10.

Patients with T2D have an increased risk of skin infection and poor wound healing, and the impaired function of keratinocytes is one of the major factors contributing to impaired wound healing in diabetes [38]. Moreover, keratinocytes also play a critical role in cutaneous innate immunity through the production of various antimicrobial peptides, having a crucial role in the defense response of skin against microbial infections [6]. Innate immunity is the first-line defense in the human body that provides non-specific defense response at all of the anatomical barriers, including the skin, and maintains a dynamic interaction with various microbes [39,40]. Patients with T2D are susceptible to infections, and the proposed mechanism is the dysregulated homeostasis of the T cell immunity with decreased innate T cells, contributing to tissue inflammation [41]. In the current study, bioinformatics tools based on different enrichment algorithms were used in order to obtain validated results of functional enrichment analysis, including singular enrichment analysis such as clusterProfiler and FunRich, gene set enrichment analysis such as GSEA and clusterProfiler, and modular enrichment analysis such as IPA, STRING, and NetworkAnalyst [26,42]. The systematic analysis using multiple tools identified consistently enriched biological pathways, including type I IFN signaling, viral defense response, and immune response among differentially expressed genes in DM-HEK cells. Among the gene clusters related to these biological functions, the results by GSEA and *z*-score estimation by IPA implicated the inhibited functions of the IFN-alpha response and antiviral response in DM-HEK cells. The results are in line with the clinical observation that patients with T2D have an increased risk of common infections [43,44].

Approximately 30% of patients with diabetes have skin changes, which are both infectious and non-infectious [45]. Various microorganisms inhabit the human skin and have essential roles in protecting against pathogens and modulating the immune system [46]. Research in skin microbiome suggests a fine balance between defense response and elicited inflammatory response in healthy skin condition, and the disturbed homeostasis is associated with many human diseases presenting cutaneous manifestations [46,47]. In the condition of dysregulated innate immunity such as T2D, alteration in the skin microbiome can result in an aberrant skin defense response and the uncontrolled colonization of pathogens, contributing to the high susceptibility to skin infection [40,48]. Our current results also indicated an altered defense response in diabetic keratinocytes, which supports the increased risk of developing skin infection related to changed bacterial diversity and aberrant skin colonization in patients with T2D [39,49].

In diabetic skin, reduced immune cell infiltrates, including Langerhans cells and dendritic cells, in the dermis and epidermis layers were observed, suggesting the potential role of altered cutaneous immunity toward an inflammatory cutaneous environment predisposing to skin wounds [5,50]. New concepts have emphasized that keratinocytes act as immune sentinels that recognize pathogens through Toll-like receptors (TLRs) to modulate predominantly innate immune response and produce type I interferons (IFNs) [6,51]. IFN-alpha belongs to the type I IFN subfamily, which signals mainly through the activation of the Janus kinase/signal transducers and activators of transcription (JAK-STAT) and mitogen-activated protein kinase (MAPK) pathways, and induces the expression of IFN-stimulated genes (ISGs) with potent antiviral activities [52,53]. Keratinocytes also produce antimicrobial peptides as a defense mechanism against pathogens when damaged [6,54]. Our current results identified the potentially inhibited functions of IFN-alpha and viral defense responses in DM-HEK cells, with TLR7/9 and IFNA1/A2 being the predicted upstream regulators affecting several downstream innate immune-related genes and ISGs, particularly *STAT1*, *IFITM1*, *ISG15*, and *MX1* (Figure 3 and Figure 7). Based on the current findings, we therefore proposed that in normal keratinocytes, responses to skin stimuli such as organism invasion and trauma may properly activate the innate immune system and trigger the immune sentinels residing in the epidermis and dermis to maintain skin homeostasis. However, in diabetic skin with an impaired function of keratinocytes, the suppressed innate immune response and reduced immune infiltrates in the dermis may result in disrupted skin homeostasis. However, the expressions of these innate immune-related genes in keratinocytes differ among various skin conditions such as atopic dermatitis and autoimmune urticaria [55,56], and the expressions of these genes in diabetic keratinocytes have not been reported. Further investigation is needed in order to confirm our current findings.

DTX3L (deltex E3 ubiquitin ligase 3L), an E3 ubiquitin ligase, belongs to the Deltex protein family, and complexes with poly(ADP-ribose) polymerase family member 9 (PARP9) to mediate the monoubiquitylation of histone and modulate the DNA damage response [57]. Research on *DTX3L* has mainly focused on its role in tumor cell growth and adhesion, including skin melanoma [58,59,60]. Based on the concept of immune advantage through enhancing the cellular response to IFN, a study by Zhang et al. suggested that the PARP9-DTX3L complex enhanced the expression of ISGs in host and promoted the degradation of viral proteases to enhance IFN-dependent immunity [61]. The dermal lymphatics provide a critical role in interstitial fluid drainage, and epidermal damage triggers immune cell activation and dermal lymphatic drainage [62]. Choi et al. proposed that laminar flow induced shear stress in lymphatic endothelial cells, and promoted lymphatic sprouting, and the DTX3L loss of function could lead to defective lymphatic sprouting [63]. In the current study, our NGS and bioinformatics results identified the down-regulated *DTX3L* and *PARP9* in DM-HEK cells, which potentially participated in IFN signaling and antiviral response, and were possibly regulated by miRNAs. Together with this evidence, we proposed the potential role of *DTX3L* in diabetic keratinocytes, which may be related to the impaired wound healing and susceptibility to skin infection of patients with T2D. Future investigation on the role of *DTX3L* in diabetic skin is necessary to provide information on its pathogenic role and advance skin care in patients with T2D.

The miRNA target prediction databases predicted miR-340-3p, which was 2.0-fold up-regulated in DM-HEK cells, as the potential upstream regulator of *DTX3L*. Studies have reported the increased expression of miR-340 in response to environmental exposures such as ultraviolet B (UVB) irradiation and fine particulate matter (PM_2.5_). With UVB irradiation, miR-340 was overexpressed in melanocyte and promoted dendrite formation and melanosome transport to neighboring keratinocytes [64]; in addition, miR-340 was also induced in retinal pigment epithelial cells to promote cell apoptosis as a possible mechanism of UVB-induced retinal damage [65]. The role of miR-340 in immune cell regulation was also reported. Elevated miR-340 in mice lung tissue after exposure to PM_2.5_ was associated with Type 1 T helper (Th1) / Type 2 T helper (Th2) cells immune imbalance [66]. In a mouse model of psoriasis, the expression of miR-340 was decreased, and treatment with miR-340 suppressed the expression of endogenous IL-17A and alleviated the clinical severity of psoriasis [67]. In conditions related to metabolic changes, circulating miR-340 was lower in maternal obesity and associated with more impaired glucose tolerance [68], and a decreased level of miR-340 was observed after a six-month high glycemic index diet [69]. In tissue regeneration, miR-340 dysregulation decreased debris removal and limited axonal growth in rat sciatic nerve crush injury [70]. The role of miR-340-3p in the accumulation of AGEs in diabetic skin or diabetic cutaneous defense response has not yet been reported. The current findings supported the potential role of miR-340-3p-*DTX3L* interaction in diabetic keratinocytes, and merit further experimental validation and clinical correlation for its potential as a diagnostic or therapeutic target in diabetic skin disorders.

There are several limitations to be addressed in the current study. Firstly, the current results were based on normal and diabetic epidermal keratinocytes isolated from single donors. To conduct a hypothesis generating study and minimize possible confounding factors between T2D populations with different glycemic control statuses, keratinocytes from single donors were used in the current in vitro study to identify the differentially expressed genes in diabetic skin. Further experimental validation is needed in order to confirm the candidate miRNA and its putative target. It is also of clinical importance to investigate the clinical specimens of different degrees of diabetic skin ulcers and at different stages of the healing process to help clarifying our current findings. Moreover, a detailed investigation of the signaling pathways that are involved in the altered skin pathology in T2D based on the mechanistic regulatory network obtained from a bioinformatics database is needed to gain a deeper understanding of the possible mechanisms that are involved in the disrupted skin homeostasis of the diabetic population.

## 5. Conclusions

Our current exploratory study indicated functionally enriched pathways of IFN-alpha signaling, viral defense response and immune response in differentially expressed genes of DM-HEK cells, with a focus on the potential role of miR-340-3p-*DTX3L* interaction in the disrupted skin homeostasis, altered the defense and immune response of diabetic skin. The findings may provide novel molecular targets in advancing diabetic skin care.

## Figures and Tables

**Table 1:** Clustered sub-networks of protein–protein interaction as determined from analysis through the Cytoscape software package with Molecular Complex Detection (MCODE).

Cluster	Score (Density *#Nodes)	Nodes	Edges	Node IDs
1	20.273	23	223	PLSCR1, IFITM1, PARP12, STAT1, IRF9, XAF1, DDX58, DHX58, OAS2, IFI44L, MX1, SP110, HERC6, PARP9, MX2, USP18, IFI44, ISG15, DDX60, EPSTI1, OAS3, IFI6, SAMD9
2	4.4	11	22	TOR4A, CXCL5, FGF2, CXCR2, LGALS3BP, CXCL1, MMP7, PPBP, MMP1, CXCL3, CXCR1
3	4	17	32	MITF, COL8A2, TEK, ITGA4, COL5A3, COL6A2, ITGB2, EDNRA, ITGAL, EGF, NTSR1, TIMP3, LYN, COL9A2, VAV1, HRH1, PLCB1
4	3.333	4	5	KDM6A, SMARCA2, NAP1L4, HIST2H2AC
5	3.333	4	5	RRAD, PRKX, RTN4RL1, LGR6
6	3	3	3	SAMHD1, HLA-A, HLA-C
7	3	3	3	GBP6, B2M, TRIM2
8	3	3	3	ALDH1A3, DIP2C, ACOXL
9	3	5	6	ACACA, CARS, DPYSL4, SHMT1, CYB5R2

**Table 2:** Top 10 biological processes enriched in the genes of cluster 1.

Biological Process	Gene Count	False Discovery Rate
Response to virus	14	7.09 × 10^−19^
Defense response to virus	13	7.09 × 10^−19^
Type I interferon signaling pathway	11	7.09 × 10^−19^
Cellular response to type I interferon	11	7.09 × 10^−19^
Defense response	16	1.10 × 10^−11^
Innate immune response	14	3.42 × 10^−11^
Immune system process	17	6.53 × 10^−11^
Immune response	15	9.23 × 10^−11^
Response to cytokine	12	2.49 × 10^−10^
Negative regulation of multi-organism process	7	9.85 × 10^−8^

**Table 3:** Potential miRNA regulations in diabetic epidermal keratinocytes.

miRNA	Fold Change (DM-HEK/N-HEK)	Putative Target	miRmap Score	TargetScan	miRDB
hsa-miR-2116-3p	2.41	DTX3L	98.87	+	−
hsa-miR-296-3p	2.30	OAS2	97.82	−	−
hsa-miR-340-3p	2.00	DTX3L	97.39	+	+
hsa-miR-4642	9.17	OAS2	97.20	−	−
hsa-miR-5010-3p	2.97	EPSTI1	97.49	+	−
hsa-miR-548b-5p	2.24	PARP9	97.23	−	+
